# Surfactin-Induced β-(1,3)-Glucan Exposure in *Aspergillus niger* Cell Wall: A Molecular Perspective

**DOI:** 10.3390/foods15040788

**Published:** 2026-02-23

**Authors:** Bo Zhang, Lingzhi Zhang, Zhengjun Pang, Wenshuo Zhang, Fenghuan Wang, Junfeng Fan, Bolin Zhang

**Affiliations:** 1 Key Laboratory of Digital-Intelligence and Dynamic Perception for Food Quality of China Light Industry, Beijing Technology and Business University, Beijing 100048, China; zhangbo@btbu.edu.cn (B.Z.); 18303559042@163.com (L.Z.); pzj461216@163.com (Z.P.); zwsdyouxiang@163.com (W.Z.); wangfenghuan@th.btbu.edu.cn (F.W.); 2 Key Laboratory of Geriatric Nutrition and Health, Ministry of Education, Beijing Technology and Business University, Beijing 100048, China; 3 School of Light Industry Science and Engineering, Beijing Technology and Business University, Beijing 100048, China; 4 Beijing Key Laboratory of Forest Food Processing and Safety, Department of Food Science and Engineering, College of Biological Sciences and Technology, Beijing Forestry University, Beijing 100083, China; fanjunfeng@bjfu.edu.cn

**Keywords:** surfactin, β-(1,3)-glucan, cell wall, binding energy, molecular dynamics simulation

## Abstract

Fruit spoilage caused by fungal pathogens jeopardizes food safety and inflicts significant economic damage. Cyclic lipopeptides (CLPs) have been applied as biofungicides by disrupting the cell membrane and intracellular components; however, the first target for antifungal action is the fungal cell wall. This study elucidates the molecular mechanism by which CLPs compromise cell wall integrity using molecular dynamics simulation and experimental validation. Among Surfactin C, Iturin A, and Fengycin A, Surfactin C exhibited the strongest binding to β-glucan (ΔE = −1970.536 kcal/mol) and the lowest free volume (7.302%), with enhanced effects at higher concentrations. Key interaction sites were identified at C=O of D-Leu3, -N-H of Leu2, and -COOH of Glu1 by Radial distribution function. In vivo assays with *Aspergillus niger* confirmed a MIC of 40 µg/mL and Surfactin-induced β-glucan exposure. FTIR and XPS analyses revealed structural reorganization and hydrogen bonding, while SEM/TEM showed spore deformation and wall rupture. These findings demonstrate that Surfactin disrupts fungal cell walls via direct complexation with β-glucan, leading to structural collapse and cell death, supporting its potential as a targeted biofungicide.

## 1. Introduction

Fungal infections are a leading cause of postharvest losses in fruits and vegetables, accounting for approximately 70% of total spoilage [1]. In recent years, cyclic lipopeptides (CLPs) have emerged as promising biofungicides for fruit preservation. Predominantly produced by *Bacillus* species via non-ribosomal peptide synthetase (NRPS) pathways, CLPs are categorized into three families, including surfactin, iturin, and fengycin. Their unique structure (a cyclic peptide linked to a fatty acid chain) confers amphiphilic characteristics and diverse biological functions, alongside favorable properties such as high efficacy, biodegradability, and low toxicity [2]. The antifungal mechanisms of CLPs are known to involve disruption of the fungal cell membrane, interference with energy metabolism, and possible binding to DNA [3,4]. However, the precise molecular targets of CLPs have not yet been fully elucidated.

The cell wall is vital in keeping fungi cell shape and establishing mechanical resistance. The absence of each cell wall component may cause the cell membrane to rupture, promoting the release of intracellular material and causing cell death [5]. The main components of the fungal cell wall are β-glucan (constitutes up to 50% of the cell wall dry mass), as well as a small amount of chitin, mannoglycoprotein and lipids. Notably, the β-Glucan forms a critical structural scaffold on the lateral wall, serving as a dynamic barrier against external stresses [6,7]. It has been established that certain antifungal agents function by inhibiting the biosynthesis of β-glucan and chitin [8]. At present, the most studied non-competitive inhibitors of β-(1,3)-glucan synthase are echinocandins and papulacandi derivatives [9]. In addition, natural bioactive compounds have been found to target β-glucanl; for example, polyacetic acid has been shown to specifically bind β-(1,3)-glucan and significantly destroy its structural integrity [10]. When antimicrobial compounds encounter the fungal cell wall, they may adhere, accumulate, and undergo structural changes or chemical modifications. Suchodolski et al. (2020) [11] found that when azole substances act on the cell wall of *Candida albicans*, they can promote β-(1,3)-glucan exposure, reduce cell hydrophobicity, and lead to cell death. Thus, the exposure of β-(1,3)-glucan appears to be an early key event in fungal cell death. This highlights the importance of investigating how CLPs contribute to β-(1,3)-glucan exposure.

Based on classical mechanics, molecular dynamics simulation establishes appropriate models and algorithms according to different objects, simulates and calculates molecular structures and properties under different systems, and obtains accurate atomic dynamics information at the atomic level [12]. Materials Studio is a computational simulation platform integrating multiple functional modules, and it builds molecular models, polymer materials and blending systems of different substances, etc., and provides scientific theoretical data for their property analysis [13].

This study introduced a novel approach by modeling CLPs and β-glucan (a critical fungal cell wall polymer) as an interactive system. Using a dual approach of molecular dynamics simulations to map binding interactions and experimental methods for verification, we deciphered the molecular mechanism of action, offering foundational support for understanding the antifungal activity of CLPs at the molecular level.

## 2. Materials and Methods

### 2.1. Materials

The strain *Aspergillus niger* (ACCC strain 30557) was obtained from the Institute of Microbiology, China Academy of Sciences (Beijing, China). The Surfactin (purity of 99.9%) and β-glucan (purity of 95.0%) were purchased from Yuanye Biotechnology Co., Ltd. Shanghai, China. PDA medium, glutaraldehyde, epoxy resin, osmium tetroxide, ethanol, acetone and lead citrate were purchased from Beijing Kebiao Biotechnology Co., LTD. Beijing, China.

### 2.2. Computational Modeling of CLPs-β-Glucan Interactions via Molecular Dynamics Simulations

#### 2.2.1. Construction and Optimization of CLPs and β-Glucan Molecular Structures

The molecular model of β-glucan was constructed using the “Visualizer” module in Materials Studio 19.0 (Accelrys Software Inc., San Diego, CA, USA). Its geometry was optimized through energy minimization to achieve the lowest-energy three-dimensional conformation [14]. Similarly, the molecular structures of Surfactin C, Iturin A, and Fengycin A were built and energetically optimized. Using the “Amorphous Cell” module, β-glucan and each cyclic lipopeptide (CLP) were packed into simulation cells at mixing ratios of 30:1 and 60:10, respectively, with an initial density set to 1 g/cm^3^. The packed systems were further relaxed to better approximate realistic conditions. Molecular dynamics simulations were then performed with the “Forcite” module under the COMPASS II force field. Each system was equilibrated successively in the isothermal–isobaric (NPT) and canonical (NVT) ensembles at 298 K. The final 500 ps of the production trajectory was extracted for analysis. Subsequently, an annealing procedure was applied by heating the system from 300 K to 500 K in 30 K intervals, followed by gradual cooling. The configuration with the lowest potential energy was selected for further structural and energetic analysis.

#### 2.2.2. Calculation of the Binding Energy

The Dreiding force field was applied for calculating the binding energy of the composite system [15]. The interaction energy ΔE (kcal/mol) between CLPs and β-Glucan was calculated using the following equation:

ΔE = E_AB_ − (E_BG_ + E_CLPs_)

where E_AB_ represents the total energy of the CLPs-β-glucan blend system, E_BG_ is the energy of β-glucan, and E_CLPs_ is the energy of CLPs.

#### 2.2.3. Calculation of Free Fraction Volume (FFV)

The system was first annealed and then equilibrated to its minimum potential energy configuration. Free volume analysis was performed by selecting solvent-accessible surfaces in the atom volumes & surfaces tool, with the free fraction volume (FFV) computed as:

FFV (%) = (Free volume)/(Free volume + Occupied volume) × 100%.

#### 2.2.4. Calculation of Radial Distribution Function (RDF)

The radial distribution function (RDF) is a powerful tool for analyzing atomic interactions. It quantifies the probability of finding an atom at a distance r from a reference atom, relative to the probability expected in a perfectly uniform distribution. This ratio inherently reflects the degree of local order and interatomic correlations within the system. A pronounced, sharp peak in the RDF signifies strong atomic ordering and close-range interaction between specific atom pairs [15]. Additionally, RDF analysis can elucidate the contribution of specific adsorption sites during the adsorption process by revealing spatial preferences and binding interactions.

### 2.3. Mechanistic Effect of Surfactin on Aspergillus niger

#### 2.3.1. Determination of Minimum Inhibitory Concentration (MIC)

PDA medium was sterilized and cooled to <50 °C before adding Surfactin to achieve final concentrations of 10, 20, 30, 40, and 60 µg/mL. After pouring into plates, 100 µL of *A. niger* spore suspension (10^6^ CFU/mL) was spread on the surface. Plates were incubated at 28 °C for 4 days. The MIC was determined as the lowest Surfactin concentration that completely inhibited visible mycelial growth.

#### 2.3.2. Cell Morphology Analysis

For both scanning electron microscopy (SEM) and transmission electron microscopy (TEM), *A. niger* spores (10^6^ CFU/mL) were cultured in 20 mL PDA medium (with 100 µL 20, 40 µg/mL Surfactin) at 28 °C for 48 h.

##### SEM

Following treatment, spores were centrifuged (6000 rpm, 15 min) and fixed overnight in 2.5% glutaraldehyde at 4 °C. After three washes with sterile water, samples were dehydrated through a graded ethanol series (30–100%, 20 min per step) and air-dried overnight. Morphology was analyzed using a JEOL JSM-IT800 SEM, Tokyo, Japan.

##### TEM

Treated spores were fixed in 2.5% glutaraldehyde, then post-fixed in 1% osmium tetroxide for 3 h at 4 °C. After dehydration in a graded acetone series (30–100%, 20 min per step), samples were infiltrated with epoxy resin and polymerized at 70 °C for 18 h. Ultrathin sections (70–90 nm) were stained with uranyl acetate and lead citrate, then examined using a Thermo Fisher Talos F200X TEM, Waltham, MA, USA (6.0 k, 80.0 kv, 2 µm).

#### 2.3.3. Exposure of β-Glucan in *A. niger* Cell Wall Induced by Surfactin

*Aspergillus niger* spores (10^6^ CFU/mL) were inoculated into Potato Dextrose Broth (PDB) and incubated at 28 °C for 10 h. The mycelia were collected, washed thoroughly with distilled water, and then treated with 0, 20, and 40 µg/mL of Surfactin for 2 h. After treatment, 0.1% aniline blue solution was added, and the mixture was incubated in the dark at 80 °C for 15 min. Fluorescence intensity was measured using a full-wavelength microplate reader, with excitation and emission wavelengths set at 398 nm and 508 nm, respectively [16].

### 2.4. Fourier Transform Infrared (FTIR) Spectroscopy

Samples of Surfactin, β-glucan, Surfactin/β-glucan mixture (1:1, *w*/*w*), *A. niger* mycelia, *A. niger* spores, and *A. niger* mycelia and spores treated with 20 µg/mL and 40 µg/mL Surfactin were subjected to lyophilization in a freeze-dryer until a constant weight was achieved and stored at −20 °C until analysis. Dried samples were mixed with KBr and pressed into pellets for FTIR spectroscopy (Alpha, Bruker, Germany) across a wavenumber range of 4000 to 400 cm^−1^. Secondary structure composition (α-helix, β-sheet, β-turn, and random coil) was quantified using PeakFit 4.12 deconvolution algorithms.

### 2.5. X-Ray Photoelectron Spectroscopy (XPS)

Prepared samples of Surfactin, β-glucan, Surfactin/β-glucan mixtures (1:1, 2:1, *w*/*w*) were freeze-dried to a constant weight. Following the method described by [17], the elemental composition and chemical states of the samples were determined using an X-ray Photoelectron Spectrometer (VG Multilab 2000, Thermo VGscientific, West Sussex, UK) operated with an excitation energy of 300 W. The obtained data were analyzed using the Advantage software, and atomic ratios of the elements were calculated based on the corresponding peak areas.

### 2.6. Data Processing and Statistical Analysis

Statistical analysis was performed using the PROC GLM procedure in SAS software (version 9.3, SAS Institute Inc., Cary, NC, USA). Differences were considered statistically significant at a *p*-value of less than 0.05. All figures were generated using Origin software (OriginLab Corporation, Northampton, MA, USA, 2018).

## 3. Results

### 3.1. Molecular Dynamics Simulation of CLPs and β-Glucan

#### 3.1.1. Interaction Energy

**Figure 1:**
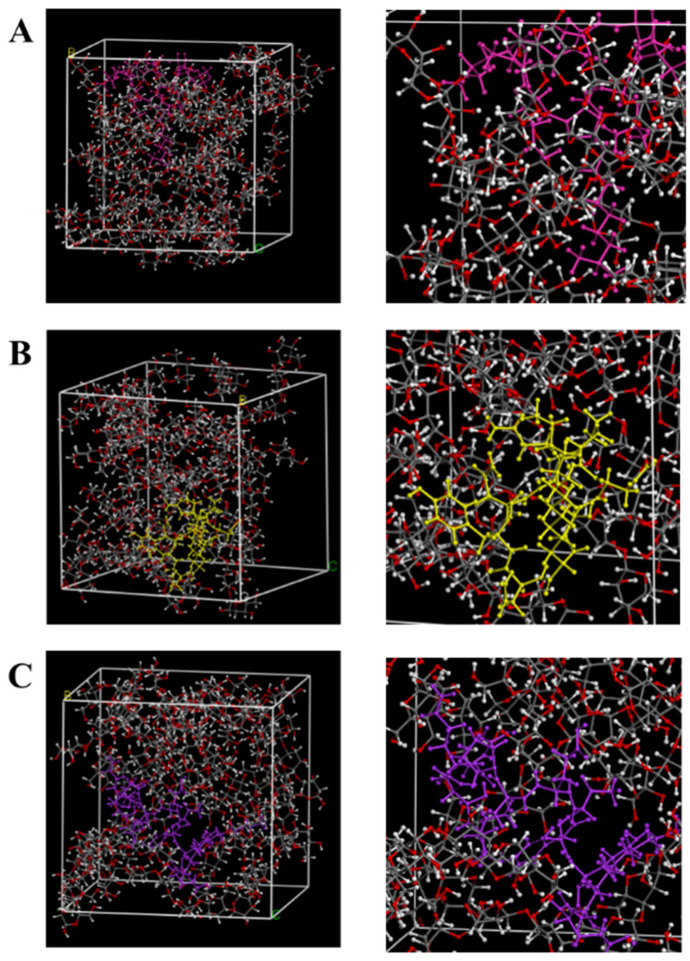
Dynamic simulation system of CLPs and β-glucan (**A**), Dynamic simulation system of Surfactin C (pink) and β-glucan; (**B**), Dynamic simulation system of Iturin A (yellow) and β-glucan; (**C**), Dynamic simulation system of Fengycin A (purple) and β-glucan.

The magnitude of the interaction energy of the blend system can be directly assessed through its interaction energy. This total interaction energy comprises valence (bonded) energy and non-bonded energy. Valence interactions primarily arise from the torsion, stretching, and bending of chemical bonds (i.e., bond lengths and angles), whereas non-bonded interactions include contributions from hydrogen bonding, electrostatic forces, and van der Waals interactions [13]. When the interaction energy ΔE is negative, substances attract each other, and the blend system exists stably. The larger the absolute value of ΔE, the stronger the interaction force. The interaction energies between different CLPs and β-glucan were calculated according to the formula, and the results are shown in Table 1. The total energy of Surfactin is 651.913 kcal/mol, including a combined bond energy of 356.573 kcal/mol and a non-bonded bond energy of 295.340 kcal/mol. The total energy ΔE_SF+BG_ of the Surfactin C and β-glucan blend system reached 8441.045 kcal/mol, with bonded and non-bonded contributions of 1931.054 kcal/mol and 6509.990 kcal/mol, respectively. For other CLPs, the computed binding energies were ΔE_IA+BG_ (−373.242 kcal/mol) for Iturin A and ΔE_FC+BG_ (−237.763 kcal/mol) for Fengycin A. The ΔE in the three blending systems was all negative, confirming the stability of each CLPs-β-glucan complex (Figure 1). The binding affinity followed the order: Surfactin > Iturin > Fengycin.

#### 3.1.2. FFV

**Figure 2:**
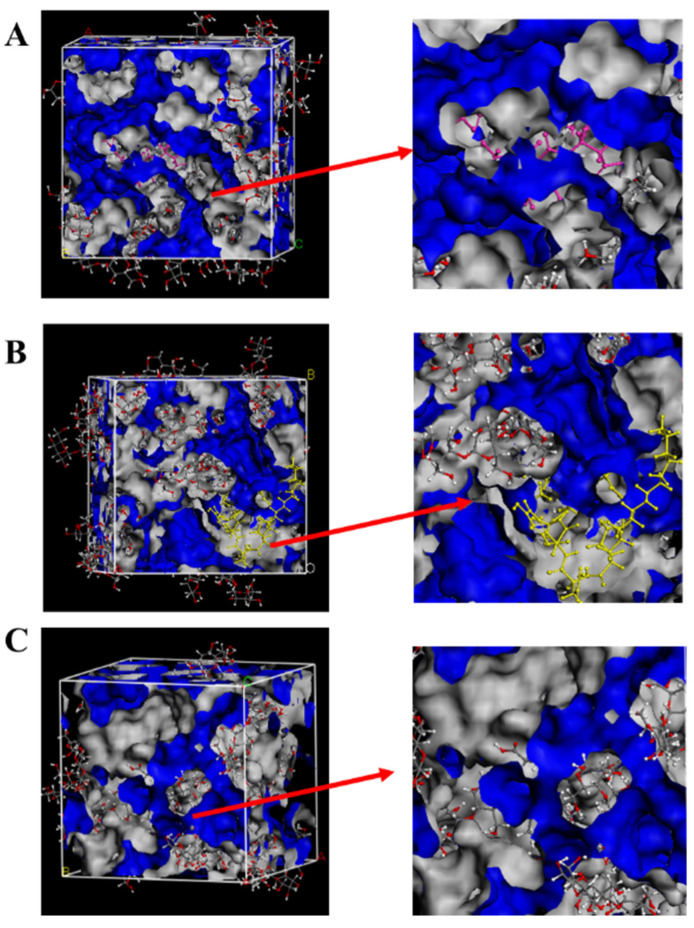
The FFV of the dynamic simulation system of CLs and β-glucan (blue region represents the free volume, the gray region represents the occupied volume). (**A**), Surfactin, pink; (**B**), Iturin, yellow; (**C**), Fengycin, purple.

The free volume theory posits that the total volume of a substance comprises both occupied volume and free volume, with molecular motion being enabled only in the presence of the latter. This concept offers a useful framework for correlating and predicting the dynamic behavior of blended systems. Figure 2 shows the spatial distribution of free volume in the blend system of CLPs and β-glucan. The free volume fraction (FFV) for blends of β-glucan with Surfactin C, Iturin A, and Fengycin A were 7.302%, 8.615%, and 9.600%, respectively. Notably, the Surfactin C–β-glucan blend exhibited the lowest FFV. Interestingly, the trend in FFV across the three systems was inversely related to their corresponding binding energies (ΔE): the higher the binding energy, the lower the FFV. It might be because the bonding energy of the Surfactin C system is very strong, and the H-bond interaction and electrostatic interaction in the non-bonding energy promote the intermolecular binding, causing the aggregation of Surfactin C and β-glucan, resulting in a decrease in the free volume fraction.

#### 3.1.3. Radial Distribution Function

**Figure 3:**
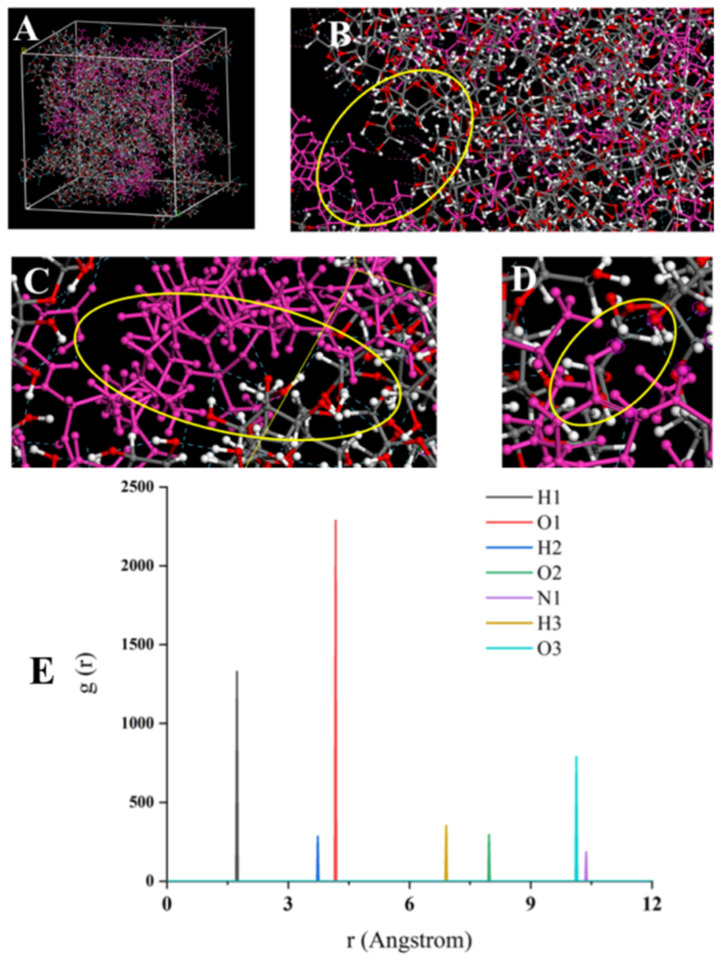
(**A**–**D**): dynamic simulation system of high concentration Surfactin and β-glucan; (**E**): the radial distribution functions (RDF) of the binding site between Surfactin and β-glucan.

To further explore the aggregation mechanism of Surfactin C and β-glucan, increasing the Surfactin C concentration to a 10:60 ratio with β-glucan (Figure 3A) resulted in a stronger binding energy (ΔE = −3212.642 kcal/mol; bond energy of −2078.512 kcal/mol; non-bonded bond energy of −1134.130 kcal/mol) and a lower free volume fraction (4.175%). As shown in Figure 3B–D, the stronger binding between Surfactin C and β-glucan formed an elaborate, interconnected network, promoting robust cross-linking. These results confirmed that higher concentrations of Surfactin C promote more effective binding and enhanced aggregation with β-glucan.

The radial distribution function (RDF) provides insight into the microstructure of a system by measuring the probability of finding atoms at a distance “r” from a reference atom. Higher probability values indicate stronger interatomic interactions, which typically arise from forces such as hydrogen bonding and van der Waals interactions. RDF analysis of the Surfactin C–β-glucan blend revealed seven distinct sharp peaks, indicating significant molecular aggregation between the two components (Figure 3E). Among these, the peaks corresponding to H1, H2, and O1 appeared at shorter distances, and the binding sites H1 and O1 were particularly high and sharp, with g(r) of 1406.18 and 2334.1, respectively, identifying them as primary binding sites during aggregation. The O3 peak was also notably high with the g(r) of 796.03, suggesting a similar contributory role. Based on the g(r) values at the seven interaction sites (Figure 3E), the binding sites were ranked in order of influence: O1 > H1 > O3 > H2 > H3 > O2 > N1. The key interaction sites were, thus, identified as the C=O of D-Leu3, the -N-H of Leu2, and the -COOH of Glu1 in Surfactin C. As indicated by the hydrogen bonds (blue dashed lines) in Figure 3D, these results confirm that hydrogen bonding is the dominant interaction between Surfactin C and β-glucan.

### 3.2. The Effect of Surfactin on Aspergillus niger In Vitro

#### 3.2.1. The β-Glucan Exposure of *Aspergillus niger* Spore

As shown in Table 2, the minimum inhibitory concentration (MIC) of Surfactin against *Aspergillus niger* was determined to be 40 µg/mL. The influence of Surfactin on β-glucan exposure in *A. niger* was further evaluated by measuring fluorescence intensity (Figure 5A). The fluorescence signal reached its highest value (4279 a.u.) at the MIC of 40 µg/mL, which was significantly stronger than that observed at lower concentrations. A concentration-dependent increase in fluorescence intensity was evident, suggesting that Surfactin compromises fungal cell wall integrity and promotes β-glucan exposure.

#### 3.2.2. Microstructure

**Figure 4:**
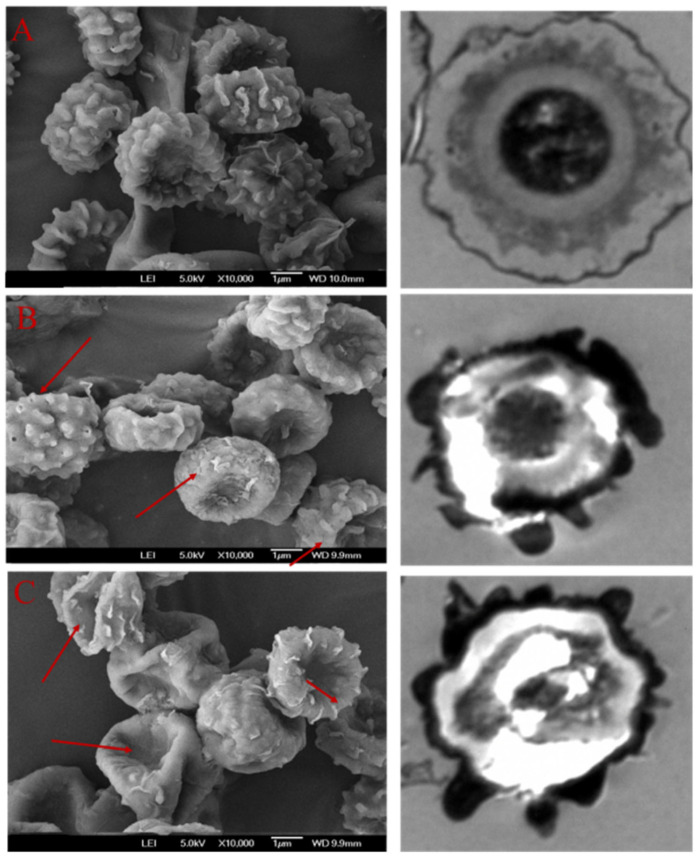
SEM and TEM of *A. niger* spore treated with Surfactin ((**A**): Control; (**B**) treated with 20 µg/mL-Surfactin, (**C**): treated with 40 µg/mL-Surfactin).

Changes in the surface morphology and ultrastructure of *Aspergillus niger* spores following Surfactin treatment were examined by SEM and TEM (Figure 4). In the untreated control, SEM revealed spores with a plump, smooth, and intact surface. Corresponding TEM images showed a full, rounded morphology, an intact cell wall, and well-organized organelles. After treatment with 20 µg/mL Surfactin, SEM observations indicated the formation of distinct pores on the spore surface, accompanied by localized peeling of the cell wall. TEM analysis further revealed that the cell wall had separated from the plasma membrane, with evidence of partial dissolution of the wall structure. At the higher concentration of 40 µg/mL Surfactin, more severe damage was observed. SEM showed pronounced surface alterations, including depressions, shrinkage, and rupture. Concurrent TEM examination confirmed extensive ultrastructural disruption: organelles were disorganized, the cell wall became thin and translucent, and the plasma membrane was compromised.

### 3.3. Analysis of the Interaction Between Surfactin and β-Glucan

#### 3.3.1. Infrared Spectroscopy

**Figure 5:**
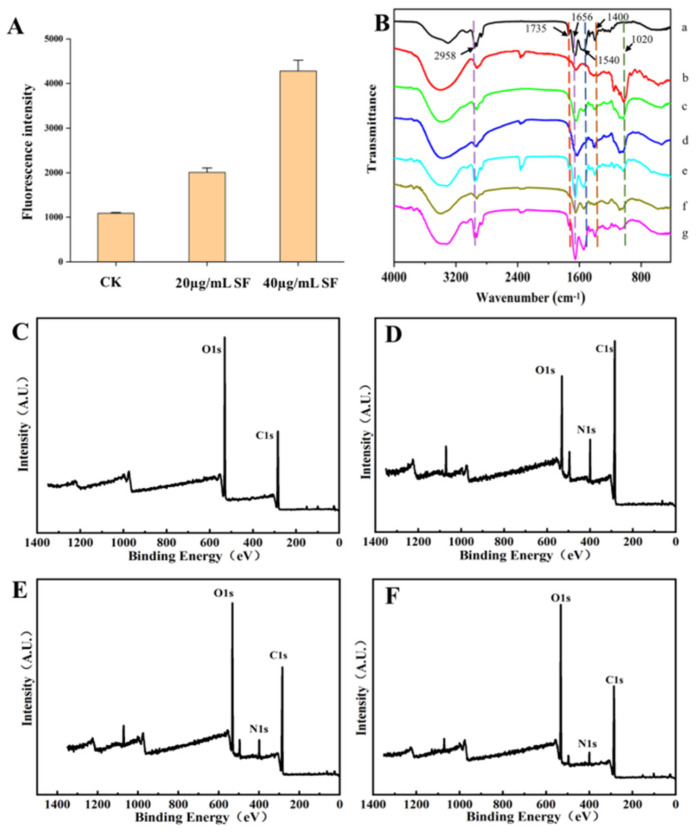
(**A**): Influence of different concentration Surfactin on β-glucan of *A. niger*; (**B**): Fourier-transform infrared spectroscopy (a: Surfactin; b: β-glucan; c: β-glucan + Surfactin (1:1); d: *A. niger* hypha; e: 40 µg/mL−Surfactin + *A. niger* hypha; f: *A. niger* spore; g: 40 µg/mL−Surfactin+ *A. niger* spore). (**C**–**F**) were the X-ray photoelectron spectroscopy analyses ((**C**): β-glucan; (**D**): Surfactin; (**E**): β-glucan + Surfactin (2:1); (**F**): β-glucan + Surfactin (1:1)).

It can be seen from Figure 5 that the main characteristic absorption peaks of Surfactin are between amide 1700–1200 cm^−1^, including amide I band (1656 cm^−1^), amide II band (1540 cm^−1^), and amide III band (1400 cm^−1^). It is mainly related to the stretching vibrations of proteins such as C=O, C=C, C=N, N=O and -COO [15], while β-glucan also conforms to the characteristic peak features of polysaccharides, with a distinct peak at 1020 cm^−1^, which is related to -COO, C-O-C and -OH in polysaccharides. After treatment with Surfactin, the 1020 cm^−1^ peak of β-glucan shifted to the right and weakened, indicating that the hydrogen bond interaction between Surfactin and β-glucan molecules was enhanced and the structure was more stable. After treatment with Surfactin and β-glucan, the asymmetric peak of C-H (2958 cm^−1^) weakened, including CH_2_ and CH_3_, indicating that the fatty acid chain of Surfactin may have a hydrogen bonding interaction with β-glucan. The amide I band (1656 cm^−1^) has a typical stretching vibration of C=O, with the peak significantly reduced. Furthermore, it was found that the peaks of the amide II band (1540 cm^−1^) and the amide III band (1400 cm^−1^) decreased and underwent a blue shift, indicating tensile deformation of C-H and C-N. Nevertheless, no new absorption peaks emerged in the infrared spectrum, indicating that Surfactin and β-glucan did not form covalent bonds.

#### 3.3.2. Secondary Structure Analysis

To further confirm the effect of Surfactin on the aggregation of β-glucan and to analyze corresponding secondary structural changes, we examined the results presented in Table 3. Upon binding to β-glucan in a 1:1 ratio, Surfactin increased the β-sheet content from 0.416 to 0.499, indicating a potential induction or stabilization of a more ordered glucan structure. More critically, when treating *A. niger* hyphae and spores with surfactin (40 µg/mL), a marked decrease in β-sheet alongside a pronounced increase in β-turn, resulting in a shift from ordered to disordered and flexible conformations. This indicates that different molecules combine with each other through intermolecular hydrogen bonds, generating many aggregates. After treatment with Surfactin, the secondary structure of *A. niger* becomes disordered. This structural disruption is attributed to an increase in β-turn content and a concurrent decrease in β-sheet content following interaction with Surfactin.

#### 3.3.3. XPS

**Figure 6:**
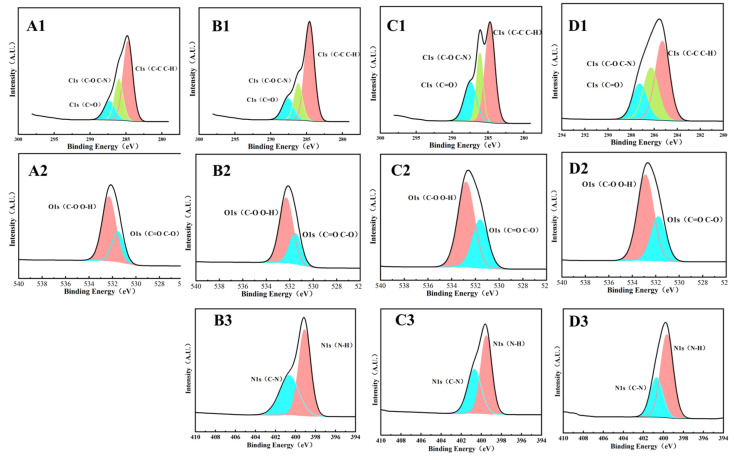
Binding energy of XPS ((**A1**,**B1**,**C1**,**D1**) are C element morphology; (**A2**,**B2**,**C2**,**D2**) are O element morphology; (**B3**,**C3**,**D3**) are N element morphology; (**A1,A2**): β-glucan; (**B1**–**B3**): Surfactin; (**C1**–**C3**): β-glucan + Surfactin (2:1); (**D1**–**D3**): β-glucan + Surfactin (1:1)).

The XPS analysis of Surfactin and β-glucan at different concentrations is presented in Figure 6. There are characteristic peaks of two components in the total spectrum. By comparing the elemental binding energies of C, H, O and N, the types of bonding between the two are explored. From Figure 6A1–C4, the spectra were charge-corrected using the C1s at 284.8 eV as an internal calibration standard. The groups were determined based on the position of the binding energy, and the relative content of the functional groups in the sample could be seen from the area of the fitted peak. It was found that after Surfactin, the content of C-N/C-H in the C spectrum increased, and the C=O decreased. The reduction of C-H/C-C bonds indicates the transformation of C-C to C-N. In the N spectrum, more N was also found to combine with C to form C-N. Correspondingly, a reduction in the C-O and O-H bond contributions was observed in the O 1s spectrum.

## 4. Discussion

The significant spoilage of fruits necessitates the development of safe, effective, and eco-friendly bio-fungicides [2]. In this context, natural antifungal CLPs of *Bacillus* spp. have emerged in recent years as a highly promising biocontrol agent. While prior antifungal research has predominantly focused on membrane interactions, the fungal cell wall serves as the first line of defense. Therefore, this study aims to evaluate the underlying molecular-level mechanisms of action targeting the cell wall.

Using molecular dynamics simulations, systems comprising three CLP families with β-glucan were established. Among these, Surfactin demonstrated the most pronounced effect, exhibiting the lowest binding energy (−1970.536 kcal/mol) and the lowest free volume fraction (FFV, 7.302%). The results revealed that greater interaction energy corresponds to a smaller free volume fraction, indicating stronger intermolecular interactions and enhanced molecular aggregation. In the case of Surfactin C, the substantial hydrogen bonding and electrostatic contributions within the non-bonded energy likely promote close association between surfactin and β-glucan molecules, thereby reducing the free volume fraction in the blended system. Thus, their association may accelerate their aggregation, leading to more exposure of β-glucan chains.

Consistent with these findings, Surfactin is reported to possess stronger biological surface activity compared with iturin and fengycin [2]. Its hydrophobic acyl chain can interact with polar heads in the cell membrane, inducing pore formation and leading to the leakage of cellular contents [18]. The strong interaction observed between Surfactin C and β-glucan molecules is therefore likely to weaken the intermolecular interactions within β-glucan itself, promoting its dissociation. This aligns with broader molecular dynamics observations; for instance, Wu et al. (2026) demonstrated that butylated hydroxytoluene more readily dissolves in solvents with stronger solute–solvent interaction energy, which enhances structural compactness, while antimicrobial peptides could interact with model bacterial membrane [12,19]. Complementary work by Cao et al. on modified asphalt revealed that increased modifier content promotes the formation of a network structure, enhances interaction energy, reduces free volume, and leads to a more compact system [13]. Similarly, the CLP polymyxin could bind irreversibly to the *E. coli* cell wall in a simple chemical environment, as shown by all-atom molecular dynamics [20]. Furthermore, higher concentrations of Surfactin favor this mechanism, as more surfactant molecules bind to β-glucan via hydrogen bonds. Key binding sites identified on Surfactin include the C=O of D-Leu^3^, the -N-H of Leu^2^, and the -COOH of Glu^1^. Together, these insights reinforce the role of Surfactin in destabilizing fungal cell walls through strong, multifactorial interactions with β-glucan.

In vivo experiments determined the MIC of surfactin against *Aspergillus niger* to be 40 µg/mL, a treatment that also enhanced the exposure of β-glucan. This aligns with prior findings, 16 µg/mL Surfactin achieved over 50% inhibition of *Candida albicans*, while an MIC of C12-Surfactin A against *Staphylococcus aureus* was 20 µg/mL [11,21]. Morphological analysis revealed that treatment with 40 µg/mL Surfactin induced pronounced changes, including obvious surface depressions, shriveling, and cracking. These effects can be attributed to the aggregation of Surfactin with β-glucan in the spore cell wall, an interaction that likely creates a structural imbalance, thins the wall, and ultimately leads to the leakage of intracellular contents. In contrast, spores treated with 20 µg/mL Surfactin did not fully rupture, a result that may be explained by the compound’s aggregation with only a portion of the cell wall at this lower concentration, generating an insufficient imbalance to cause complete lysis.

Surfactin-treated spores exhibited severe ultrastructural damage, including disorganized organelles, a thinned and translucent cell wall, and a compromised plasma membrane. These observations suggest that at higher concentrations, Surfactin extensively aggregates with the cell wall, promoting cytoplasmic leakage and ultimately cell death. Supporting this target-specific action, Wang et al. investigated the antifungal effect of Surfactin on *Aspergillus niger* and observed that while the treated hyphae became shriveled and wilted, the spore head was severely collapsed [22]. And a novel CLP baelezcin A inhibited the gray mold by dispersing the spore and hyphae [23]. Similarly, dectin-1 mediated the recognition of β-glucan in *Candida albicans*, functioning as a high-affinity receptor critical for antifungal immune responses [16]. Drawing a parallel to this mechanism, it is plausible that Surfactin inhibits *A. niger* by acting as a high-affinity ligand or molecular disruptor that specifically targets β-glucan. By interfering with the intermolecular associations of β-glucan, Surfactin may increase its exposure and compromise the structural integrity sustained by the cell wall. This progressive degradation of cellular morphology ultimately leads to the loss of viability in *A. niger*.

According to FTIR analysis, after treatment with Surfactin, the 1020 cm^−1^ peak of β-glucan shifted to the right and weakened, indicating that the hydrogen bond interaction between Surfactin and β-glucan molecules was enhanced and the structure was more stable. In secondary structure analysis, the random curling decreases, while the β-folding and α-helical structures increase, making the secondary structure more ordered. This indicated that different molecules combine with each other through intermolecular hydrogen bonds, generating aggregates. Otherwise, Zn^2+^ enhanced the hydrophobic and intermolecular electrostatic interactions of CLPs by binding to the -N-H, -COOH, and -C-O groups, increasing the exposure of hydrophobic groups and β-folding [24]. And the unique structure of head-tail CLPs may enhance the antifungal activity, like EeCentrocin 1 [25]. Consequently, these functional groups are key targets for further investigation. Notably, the XPS results revealed that after treatment with Surfactin, the conversion from C-C to C-N bonds was observed, alongside a decrease in C-O/O-H functional groups. These changes suggest that the O-H groups of β-glucan likely form hydrogen bonds with the C=O groups of Surfactin, and that C atoms of β-glucan may undergo cross-linking or aggregation with the amino groups on Surfactin. These findings are consistent with the results obtained from both infrared spectroscopy and molecular dynamics simulations. However, when β-glucan was blended with either high or low concentrations of Surfactin, no significant change was detected in the content of N-H bonds. This may be attributed to the transformation of intramolecular N-H bonds within Surfactin into intermolecular N-H bonds between Surfactin and β-glucan, aligning with the radial distribution function analysis derived from the molecular dynamics simulations.

Overall, the molecular dynamics simulations predicted a strong binding affinity between Surfactin and β-glucan. Driven by hydrogen bonding and electrostatic interactions, surfactin exhibited a pronounced propensity for molecular self-assembly. Experimental validation showed that treatment with Surfactin caused severe morphological damage, including concave depressions on the spore surface, wall thinning, and leakage of cellular contents. Spectroscopic analysis confirmed these interactions, indicating enhanced hydrogen bonding and potential cross-linking between functional groups of Surfactin (C=O, -;NH_2_, -COOH) and β-glucan. Together, molecular dynamics simulations and experimental validation [26] provide a new perspective on the antifungal mechanism of Surfactin, thereby revealing a unique molecular-level strategy that targets the fungal cell wall.

## 5. Conclusions

This study demonstrates that Surfactin exerts its antifungal activity primarily by targeting and destabilizing the structural integrity of the fungal cell wall through specific, high-affinity interactions with β-glucan. Molecular dynamics simulations revealed Surfactin’s superior binding capacity to β-glucan, characterized by the lowest binding energy and reduced system free volume, indicative of strong intermolecular aggregation driven by hydrogen bonding and electrostatic forces. Experimental validation showed that 40 µg/mL Surfactin treatment led to severe morphological damage, including spore surface depression, wall thinning, and content leakage, consistent with the simulated disruption of β-glucan’s intermolecular network. Key interaction sites were identified at C=O of D-Leu3, -N-H of Leu2, and -COOH of Glu1. This study not only provides a theoretical foundation for the application of Surfactin as a biofungicide but also offers novel insights into the mechanisms of antifungal action.

## Figures and Tables

**Table 1:** Interaction force of the dynamic simulation system of CLPs and β-glucan (SF: surfactin; BG: β-glucan; IA: iturin; FC: fengycin).

CLPs	Simulation System	Total Energy (kcal/mol)	Valence Energy (kcal/mol)	Non-Bond Energy (kcal/mol)
Surfactin	E_SF_	651.913	356.573	295.340
	E_BG_	9759.668	4405.263	5354.405
	E_SF+BG_	8441.045	1931.054	6509.990
	ΔE_SF+BG_	−1970.536	−2830.791	860.255
Iturin	E_IA_	135.777	123.582	12.195
	E_BG_	4040.383	29.455	4010.928
	E_IA+BG_	3802.918	92.316	3710.602
	ΔE_IA+BG_	−373.242	−60.721	−312.521
Fengycin	E_FC_	55.294	82.623	−27.329
	E_BG_	3269.565	−220.697	3490.262
	E_FC+BG_	3087.096	−260.794	3347.890
	ΔE_FC+BG_	−237.763	−122.72	−115.043

**Table 2:** Inhibition of different concentration Surfactin on *A. niger*.

Samples	Inhibition (%)
CK	0
10 µg/mL Surfactin	45
20 µg/mL Surfactin	60
30 µg/mL Surfactin	87
40 µg/mL Surfactin	100
60 µg/mL Surfactin	100

**Table 3:** Secondary structure analysis.

Samples	β-Sheet	Random Coil	α-Helix	β-Turn
Surfactin	0.1657	0.1532	0.1606	0.5204
β-1,3-glucan	0.4162	0.2454	0.2115	0.1269
β-1,3-glucan + Surfactin (1:1)	0.4998	0.1497	0.2330	0.1873
*A. niger* hypha	0.4302	0.1161	0.2122	0.2412
Surfactin (40 µg/mL) + *A. niger* hypha	0.3428	0.1099	0.1125	0.4347
*A. niger* spore	0.3612	0.1404	0.1393	0.3590
Surfactin (40 µg/mL) + *A. niger* spore	0.2175	0.1478	0.1605	0.4741

## Data Availability

The data that support the findings of this study are available from the corresponding author upon reasonable request.

## References

[1] Al-Obadi M., Ayad H., Pokharel S., Ayari M.A. (2022). Perspectives on food waste management: Prevention and social innovations. Sustain. Prod. Consum..

[2] Zhang B., Xu L.L., Ding J.L., Wang M.Z., Ge R., Zhao H.F., Zhang B.L., Fan J.F. (2022). Natural antimicrobial lipopeptides secreted by *Bacillus* spp. and their application in food preservation, a critical review. Trends Food Sci. Technol..

[3] Bakker C., Graham H.R., Popescu I., Li M., McMullin D.R., Avis T.J. (2024). Fungal membrane determinants affecting sensitivity to antifungal cyclic lipopeptides from *Bacillus* spp. Fungal Biol..

[4] Liu Y.N., Lu J., Sun J., Lu F.X., Bie X.M., Lu Z.X. (2019). Membrane disruption and DNA binding of *Fusarium graminearum* cell induced by C16-fengycin A produced by *Bacillus amyloliquefaciens*. Food Control.

[5] Ruiz-Herrera J., Ortiz-Castellanos L. (2019). Cell wall glucans of fungi. A review. Cell Surf..

[6] Zhang L.B., Li Y.T., Yang Z.H., Cui Z., Guan Y., Liu G.H. (2026). Elucidating the role of alg14 in governing cell wall architecture and biological functions in the entomopathogenic fungus *Beauveria bassiana*. Pestic. Biochem. Physiol..

[7] Curto M.N., Butassi E., Ribas J.C., Svetaz L.A., Cortés J.C.G. (2021). Natural products targeting the synthesis of β-(1,3)-D-glucan and chitin of the fungal cell wall. Existing drugs and recent findings. Phytomedicine.

[8] Romero I., Sanchez-Ballesta M.T., Maldonado R., Escribano M.I., Merodio C. (2006). Expression of class I chitinase and β-1,3-Glucanase genes and postharvest fungal decay control of table grapes by high CO_2_ pretreatment. Postharvest Biol. Technol..

[9] Healey K.R., Perlin D.S. (2018). Fungal resistance to echinocandins and the MDR phenomenon in *Candida glabrata*. J. Fungi.

[10] Lee K.K., Kubo K., Abdelaziz J.A., Cunningham I., DeSilva D.A., Chen X., Okada H., Ohya Y., Gow N. (2018). Yeast species-specific, differential inhibition of β-1,3-glucan synthesis by poacic acid and caspofungin. Cell Surf..

[11] Suchodolski J., Derkacz D., Muraszko J., Panek J.J., Jezierska A., Łukaszewicz M., Krasowska A. (2020). Fluconazole and lipopeptide surfactin interplay during *Candida albicans* plasma membrane and cell wall remodeling increases fungal immune system exposure. Pharmaceutics.

[12] Wu X., Cheng X.W., Sun S.Y., Ni J.X., Han Y., Qu M.W., Liu B.S., Sun H. (2026). Deciphering the butylated hydroxytoluene dissolution behavior in solid-liquid systems through coupled experimental and molecular dynamic simulation approaches. Food Chem..

[13] Cao X.J., Yi L., Ding Y.J., Su Y. (2022). Study on the influence of micro action of modifier content on modified asphalt based on molecular dynamics simulation. New Chem. Mater..

[14] Han Y.X., Deng S.Y., Fu J., Wang B., Gao J., Nie M., Wu Q.J., She Y.H., Zhang F. (2025). Green synthesis and functionalization of rice husk-derived SiO_2_ nanoparticles for scale inhibition: A multiscale study combining experiments, molecular dynamics simulations, and quantum chemical calculations. Chem. Eng. J..

[15] Zhang H., Xu Z.M., Zhao Y., Wang J.T., Wang B.B. (2024). Inhibition of calcium carbonate nucleation and crystallization by carboxymethyl dextran: Experiments and molecular dynamics simulations. Desalination.

[16] Hu T.L., Shi G.X., Xu Z.Q., Duan Q.J., Shao J., Wang T.M., Wu D.Q., Wang C.Z. (2018). Inhibitory effect of Butyl alcohol extract of BaiTouWeng decoction on *Candida albicans* cell wall. Chin. J. Mycol..

[17] Alwi M.A.M., Ahmad M.N., Kayed S.F., Dzulkifli N.N., Abu Samah M.A., Pauzi H., Normaya E. (2026). Structural elucidation of a novel dual-substituted thiosemicarbazone scaffold as an efficient copper corrosion inhibitor: Insights from RSM, XPS, and DFT-Fukui analyses. J. Mol. Struct..

[18] Falardeau J., Wise C., Novitsky L., Avis T.J. (2013). Ecological and mechanistic insights into the direct and indirect antimicrobial properties of *Bacillus subtilis* lipopeptides on plant pathogens. J. Chem. Ecol..

[19] Bogdanova L., Valiullina Y., Faizullin D., Kurbanov R.K., Ermakova E. (2020). Spectroscopic, zeta potential and molecular dynamics studies of the interaction of antimicrobial peptides with model bacterial membrane. Spectrochim. Acta Part A.

[20] Smith S.P.I., Pedebos C., Khalid S. (2024). Molecular crowding alters the interactions of polymyxin lipopeptides within the periplasm of *E. coli*: Insights from molecular dynamics. J. Phys. Chem. B.

[21] Wu Y.D. (2020). Optimization of Surfactin Production and Antibacterial Effect Research. Master’s Thesis.

[22] Wang Q.Y., Lin Q.L., Peng K., Cao J.D., Yang C., Xu D. (2017). Surfactin variants from *Bacillus subtilis* natto CSUF5 and their antifungal properties against *Aspergillus niger*. J. Biobased Mater. Bioenergy.

[23] Yuan Z.Q., Zhou B., Wu X.N., Wang L., Li G.L., Liu J.W., Kang Q.J., Wu D.R., Li J. (2023). Isolation, structural characterization and sporicidal properties of Baelezcin A, a novel cyclic lipopeptide from *Bacillus velezensis* SJ100083 against gray mold. J. Sci. Food Agric..

[24] Ding J.L., Zeng S.F., Wang Y.Q., Yin X.Y., Zhang B., Zhang B.L., Xu S.D., Zhang Y.Y., Zheng J.F., Fan J.F. (2023). Metal coordinating-induced self-assembly of cyclic lipopeptides into high-performance antimicrobial supramolecules. Food Chem..

[25] Simonović D., Dey H., Johansen N., Anderssen T., Hansen I.K.Ø., Devold H., Vasskog T., Blencke H.-M., Øyen F.J., Fredheim E.G.A. (2025). Antimicrobial activity of short analogues of the marine peptide EeCentrocin 1: Synthesis of lipopeptides and head-to-tail cyclic peptides and mechanism of action studies. J. Pept. Sci..

[26] Chen Y.F., Mao J.S., Wang B.G., Wang C.G. (2025). Performance characterization of surface-coated ultrafine hexanitrostilbene-IV by experiment and simulation. J. Mol. Model..

